# Passive smoking exposure and the risk of hypertension among non-smoking adults: the 2015–2016 NHANES data

**DOI:** 10.1186/s40885-020-00159-7

**Published:** 2021-01-01

**Authors:** Onoja Matthew Akpa, Akinkunmi Paul Okekunle, Jeffery Osahon Asowata, Babatunde Adedokun

**Affiliations:** 1 Department of Epidemiology and Medical Statistics, College of Medicine, University of Ibadan, PMB 900001 UI Post Office, Ibadan, 200284 Nigeria; 2 Center for Genomic and Precision Medicine, College of Medicine, University of Ibadan, Ibadan, 200284 Nigeria; 3 Preventive Cardiology Research Unit, Institute of Cardiovascular Diseases, College of Medicine, University of Ibadan, Ibadan, 200284 Nigeria; 4 The Postgraduate College, University of Ibadan, PMB 900001, Ibadan, Nigeria; 5 Nutritional Epidemiology Lab, Department of Food and Nutrition, College of Human Ecology, Seoul National University, 1 Gwanak-ro, Gwanak-gu, Seoul, IL 08826 Korea; 6 Research Institute of Human Ecology, Seoul National University, 1 Gwanak-ro, Gwanak-gu, Seoul, IL 08826 Korea; 7 Center for Clinical Cancer Genetics and Global Health, Department of Medicine, University of Chicago, Chicago, IL 60637 USA

**Keywords:** Passive smoke exposure, Hypertension, NHANES

## Abstract

**Background:**

Hypertension is a major public health problem and a prominent risk factor for cardiovascular diseases. However, whether passive smoking exposure (PSE) is associated with the risk of hypertension is scarcely understood. This study assessed the association between PSE and the risk of hypertension among adults (≥18 years) in the United States of America.

**Methods:**

Three thousand and sixty-seven adults were identified from the 2015–2016 National Health and Nutrition Examination Survey and the association between PSE and hypertension (adjusting for relevant confounders) was examined using multivariable adjusted-logistic regression analysis at *P* < 0.05.

**Results:**

Mean age of respondents was 46.5 ± 17.9 years. Overall, 23.7% of respondents reported PSE and 32.6% were hypertensives (of which only 14.3% were aware of their hypertensive state) Also, adjusted odds of hypertension for participants with PSE was 1.038 (1.037, 1.040), *P* < 0.0001, in the overall population. Also, PSE aggravated odds of hypertension among young adults – < 60 years (aOR: 1.095, 95CI%: 1.094 to 1.097), *P* < 0.0001, and old adults – ≥60 years (aOR: 1.110, 95% CI: 1.108 to 1.113), *P* < 0.0001. Similarly, PSE was associated with increased odds of hypertension among women (aOR: 1.240, 95% CI: 1.238 to 1.242), *P* < 0.0001 but not among men (aOR: 0.755, 95% CI: 0.754 to 0.757), *P* < 0.0001.

**Conclusions:**

PSE was independently associated with the risk of hypertension particularly among women, young and old adults. A multi-ethnic longitudinal cohort may help ascertain causality and provide more evidence for appropriate interventions.

**Supplementary Information:**

The online version contains supplementary material available at 10.1186/s40885-020-00159-7.

## Background

Hypertension is a major public health problem worldwide [1] and a chronic disorder with tendencies to promote cardiovascular diseases (CVD) [2]. Recent estimates revealed about one in every three adults in the United States (US) had hypertension in 2015 [3] with a sudden surge to 45.4% in 2018 [4]. It accounts for 23.1% of all mortality in the US between 2000 and 2013 [5] in addition to an estimated compelling cost of care of about 51.2 billion US dollars between 2012 and 2013 [6]. Several reports [6–12] have demonstrated some potential risk factors for hypertension but with limited information on passive smoking exposure (PSE).

PSE can be defined as the exposure of an individual(s) to sidestream and mainstream smoke arising from burning cigarettes and/or exhaled by a smoker [13]. It accounted for over nine million disability life adjusted years and 331, 000 deaths in 2013 globally [14]. The widespread burden of PSE necessitates discerning the potential contribution(s) of PSE to the onset of chronic diseases.

Whether PSE is sufficient to trigger pathological alterations in the vasculature and myocardium to promote elevated blood pressure perpetually remains unclear. This understanding is likely to provide novel insights into the significance of PSE in chronic diseases, guide public health interventions to alleviate the burden of PSE and promote suitable blood pressure control management strategies for exposed population(s). Besides, the multiracial background of the US population renders it a suitable cohort to test the hypothesis of this study and perhaps simplify its findings across populations.

The present analysis assessed the association between PSE and the risk of hypertension using the 2015–2016 National Health and Nutrition Examination Surveys (NHANES). Similarly, we tested whether the PSE and risk of hypertension relationship differed by age (between younger adults - < 60 years and old adults - ≥60 years) and by sex in the same population sample.

## Methods

### Study design, sampling strategies and participants

The 2015–2016 NHANES is a population-based survey conducted by the National Center for Health Statistics (NCHS) of the Centre for Disease Control and Prevention (CDC) in the United States. It is a cross-sectional and nationally representative data of non-institutionalized civilian residents of the United States across 50 states and Washington DC sampled through a multistage probability design [15]. Of the 9971 respondents who were interviewed, we excluded 3979 respondents age < 18 years, 70 pregnant women, 2851 smokers and 4 respondent with missing information on smoking and hypertension. The final analysis was based on 3067 respondents (1961 women and 1106 men). All participants provided written informed consent, and the survey protocols were approved by the NCHS Ethics review board.

### Data collection and definition of phenotypes

Data collection in the NHANES data involved the use of standardized instruments administered at home by trained personnel followed by a comprehensive physical checkup at a mobile examination centre [15]. Also, details of the instrument and methods for data collection; questionnaires, data coding, documentation and procedure manual have been reported elsewhere [16]. Information on the sex of respondents was self-reported as male and female. Age was reported in years and classified as < 60 years (young adults) and ≥ 60 years (old adults). Ethnicity was self-reported and categorized as Hispanics only, White only, Black only and other races. Also, respondents’ education was reported from never to tertiary and classified as <High School (where respondents reported completing less than 9th grade as the highest level of education) and ≥ High School (if respondents reported completing formal education – at least 9th grade).

Employment status was defined as ‘yes’ where respondents affirmatively asserted a current involvement in any form of a paid job, otherwise unemployed. Annual household income was *≤ $24,999 or > $24,999.* Marital status was self-reported and classified as never married, married/living with a partner and widowed/divorced/separated. Alcohol use was defined as the reportage of a minimum of 12 drinks of any form of alcoholic drink in the past year or a lifetime. Similarly, respondents were classified as a habitual smoker if they affirmatively assented to smoking ≥100-lifetime cigarettes or current use of any form of tobacco products or e-cigarette. Weight (in kg) and height (in cm) of respondents were measured by trained medical personnel using a standard protocol [17] and body mass index (BMI) was estimated [as a function of weight (kg) divided by square of height (m^2^)] and classified using World Health Organization guidelines [18].

### Passive smoking exposure (PSE) – the predictor variable

In the NHANES data, respondents were requested to provide information on whether they had worked at a job or spent time in a restaurant/bar/car/indoor area where someone smoked indoors in the last 7 days. In this study, PSE was defined as affirming to being exposed to smoke arising from any form of cigarette in any indoor area such as; the household, restaurant, bar, car, etc. [19, 20].

### Definition of hypertension – the outcome variable

Systolic and diastolic blood pressure (BP) measurements were taken at the mobile examination centres by trained medical personnel using a standard protocol [15, 21]. Three consecutive BP readings were taken by the auscultatory method (after respondents are rested in a sitting position for 5 min) using a mercury sphygmomanometer and suitable cuff dimension based on upper arm circumference measurement. Where BP readings were interrupted a fourth reading was taken. All available BP readings were used to determine the mean systolic-BP (SBP) and diastolic-BP (DBP) for each respondent [22]. Hypertension was defined as mean SBP ≥ 140 mmHg or DBP ≥ 90 mmHg or/and use of anti-hypertensive or blood pressure-lowering drugs according to the American Heart Association guidelines [23]. To discern awareness of hypertension, respondents were asked if they have been told by a doctor or medical personnel that they had hypertension, also referred to as high blood pressure [22].

### Statistical analysis

Respondents’ characteristics were stratified by hypertension status (no/yes) and compared using the Chi-square (*χ*2) test or independent sample *t-*test for categorical or continuous data respectively. We further stratified our analyses by age (< 60 years and ≥ 60 years) and sex (men and women) to evaluate the potential contribution of ageing and sex difference on these analyses. Multivariable logistic regression analysis was utilized to estimate the adjusted odds ratio (aOR) and 95% confidence interval (CI) for the association between PSE and hypertension (adjusting for relevant confounders) in the entire sample, stratified by age groups and sex. To determine whether PSE was dose-dependently associated with hypertension, we combined the entire dataset and stratified the population as “No PSE and non-smokers only’”, “PSE exposure and non-smokers only”, “smokers only” and “PSE exposure and smokers only” and multivariable logistic regression analysis was utilized to estimate the aOR and 95% CI for the dose-dependent relationship between PSE and hypertension (adjusting for relevant confounders). All estimates in this reported were weighted using the CDC recommendations  to avoid confounding attributable to oversampling, non-response, disproportionate population coverage and ensure our estimates are nationally representative of the US population [15]. All statistical analyses were computed at a statistical significance of two-sided *P* < 0.05 using SPSS for Windows (version 21); IBM Corporation.

## Results

### Characteristics of participants

The characteristics of respondents are presented in Table 1. Overall, the mean age was 46.5 ± 17.9 years, 63.3% were women while 74.7% of the participants were <  60 years. Also, 57.1% were Whites, 64.3% were employed and 66.0% were married. Most participants reported having an education less than high school (96.6%) and an annual household income *>$24,999* (83.0%). Also, 74.8% reported using any form of alcoholic drink in the past year and 35.5% were obese. Similar trends were observed in the age strata, but the mean age among young adults was 38.3 ± 12.2 years. Also, the mean age was 70.9 ± 6.5 years and 28.4% were employed among old adults.

**Table 1:** Socio-demographic and metabolic characteristics of all respondents according to Hypertension status in the NHANNES Data

Characteristics	All (***N*** = 3067)				< 60 years (***n*** = 2213)				≥60 years (***n*** = 854)			
	Hypertension Status				Hypertension Status				Hypertension Status			
	Combined	No	Yes	***P***	Combined	No	Yes	***P***	Combined	No	Yes	***P***
Sex												
*Women*	1961(63.3)	1241(66.7)	720(33.3)	< 0.0001	1386(61.2)	1089(81.1)	297(18.9)	< 0.0001	575(69.5)	152(29.2)	423(70.8)	< 0.0001
*Men*	1006(36.7)	707(68.5)	399(31.5)		827(38.8)	619(77.1)	208(22.9)		279(30.5)	88(36.2)	191(63.8)	
Age (years)	46.5 ± 17.9	40.2 ± 15.7	59.6 ± 15.0	< 0.0001	38.3 ± 12.2	36.2 ± 11.8	46.3 ± 9.9	< 0.0001	70.9 ± 6.5	70.1 ± 6.2	71.3 ± 6.6	< 0.0001
*< 60 years*	2213(74.7)	1708(79.6)	505(20.4)	< 0.0001								
*≥ 60 years*	854(36.7)	240(31.3)	614(68.7)									
Race												
*White only*	764(57.1)	479(66.9)	285(33.1)	< 0.0001	451(51.6)	378(82.9)	73(17.1)	< 0.0001	313(73.4)	101(33.6)	212(66.4)	< 0.0001
*Hispanics only*	1067(18.7)	673(71.9)	394(28.1)		780(21.7)	605(79.6)	175(20.4)		287(9.8)	68(21.7)	219(78.3)	
*Black only*	633(12.5)	360(60.5)	273(39.5)		496(14.0)	332(67.9)	164(32.1)		137(7.9)	28(21.6)	109(78.4)	
Others	603(11.7)	436(69.8)	167(30.2)		486(12.7)	393(78.7)	93(21.3)		117(8.9)	43(32.2)	74(67.8)	
Education												
*< High School*	2886(96.6)	1779(66.4)	1107(33.6)	< 0.0001	2035(95.4)	1539(78.8)	496(21.2)	< 0.0001	851(99.9)	240(31.4)	611(68.6)	< 0.0001
*≥ High School*	178(3.4)	166(95.3)	12(4.7)		175(4.6)	166(95.9)	09(4.1)		03(0.1)	00(0.0)	03(100.0)	
Employed												
*No*	1255(35.7)	646(54.2)	609(45.8)	< 0.0001	622(23.6)	482(79.9)	140(20.1)	< 0.0001	633(71.6)	164(67.1)	469(70.9)	< 0.0001
*Yes*	1804(64.3)	1295(74.6)	509(25.4)		1585(76.4)	1221(79.4)	364(20.6)		219(28.4)	74(36.0)	145(64.0)	
Income^a^												
*≤ $24,999*	740(17.0)	402(57.8)	338(42.2)	< 0.0001	444(14.4)	335(78.6)	109(21.4)	< 0.0001	296(25.0)	67(21.2)	229(78.8)	< 0.0001
*> $24,999*	2073(83.0)	1390(69.9)	638(30.1)		1604(85.6)	1245(79.7)	359(20.3)		469(75.0)	145(35.5)	324(64.5)	
Marital Status												
*Never married*	520(17.8)	408(83.0)	112(17.0)	< 0.0001	472(22.5)	398(87.1)	74(12.9)	< 0.0001	48(4.7)	10(27.8)	38(72.2)	< 0.0001
*Married*^b^	1805(66.0)	1150(67.9)	655(32.1)		1343(68.8)	999(77.6)	344(22.4)		462(58.1)	151(35.6)	311(64.4)	
*Widowed*^c^	563(16.2)	221(41.8)	342(58.2)		219(8.7)	142(67.0)	77(33.0)		344(37.2)	79(25.2)	265(74.8)	
Alcohol use												
*No*	847(25.2)	512(65.9)	335(34.1)	< 0.0001	555(23.9)	438(81.9)	117(18.1)	< 0.0001	292(28.9)	74(28.6)	218(71.4)	< 0.0001
*Yes*	1773(74.8)	1104(66.5)	669(33.5)		1303(76.1)	978(78.1)	325(21.9)		470(71.1)	126(31.8)	344(68.2)	
BMI (kg/m^2^)	28.9 ± 7.1	27.6 ± 6.4	31.5 ± 7.7	< 0.0001	28.7 ± 7.0	27.7 ± 6.5	32.5 ± 7.6	< 0.0001	29.5 ± 7.2	27.2 ± 5.3	30.5 ± 7.7	
Normal Weight	882(32.2)	691(81.6)	191(18.4)	< 0.0001	691(34.2)	618(92.0)	73(8.0)	< 0.0001	191(26.3)	73(42.0)	118(58.0)	< 0.0001
Overweight	914(32.0)	581(66.9)	333(33.1)		631(31.1)	505(79.1)	126(20.9)		283(34.5)	76(34.2)	207(65.8)	
Obese	1104(35.8)	559(55.1)	545(44.9)		738(34.7)	494(67.7)	289(32.3)		321(39.2)	65(22.3)	256(77.7)	
Hypertension Awareness												
*No*	2106(72.0)	1984(93.5)	158(6.5)	< 0.0001	1797(83.5)	1708(95.4)	89(4.6)	< 0.0001	309(38.4)	240(81.7)	69(18.3)	< 0.0001
*Yes*	960(28.0)	00(0.0)	960(100.0)		416(16.5)	00(0.0)	416(100.0)		544(61.6)	00(0.0)	544(100.0)	
SBP (mmHg)	122.4 ± 17.2	115.4 ± 10.7	136.4 ± 19.1	< 0.0001	118.4 ± 14.4	114.7 ± 10.5	132.7 ± 17.9	< 0.0001	133.8 ± 19.4	120.9 ± 11.1	139.7 ± 19.5	< 0.0001
DBP (mmHg)	69.8 ± 11.1	68.7 ± 9.0	72.0 ± 14.1	< 0.0001	71.1 ± 9.9	69.2 ± 8.7	78.2 ± 11.0	< 0.0001	66.1 ± 13.2	64.8 ± 10.3	66.7 ± 14.4	< 0.0001
PSE												
*No*	2265(76.3)	1412(67.1)	853(32.9)	< 0.0001	1574(74.8)	1214(80.3)	360(19.7)	< 0.0001	691(80.9)	198(31.2)	493(32.4)	< 0.0001
*Yes*	795(23.7)	532(68.2)	263(31.8)		636(25.2)	491(77.4)	145(22.6)		159(19.1)	41(68.8)	118(67.6)	

^a^ - annual household income, ^b^ - married/living with partner, ^c^ - widowed/divorced/separated

Continuous variables are presented as mean ± standard deviation and compared using the t-test

Categorical variables are presented as n(%) and compared using the *x*^2^ test

Findings of sex-stratification in the population are presented in Table 2. Among women only, mean age and BMI was 48.0 ± 18.1 years and 29.3 ± 7.5 kg/m^2^ respectively. Also, 27.8% were old adults, 72.6% reported using alcohol, 30.2% reported being aware they had hypertension and 38.4% were obese. Among men only, mean age was 44.0 ± 17.4 years, mean BMI was 28.2 ± 6.8 kg/m^2^, 21.0% were old adults, 78.3% reported using alcohol, 24.1% reported being aware they had hypertension and 31.3% were obese.

**Table 2:** Socio-demographic and metabolic characteristics of all respondents according to Hypertension status stratified by sex of respondents in the NHANNES Data

Characteristics	Women (***N*** = 1961)				Men (***n*** = 1006)			
	Hypertension Status				Hypertension Status			
	Combined	No	Yes	***P***	Combined	No	Yes	***P***
Age (years)	48.0 ± 18.1	41.2 ± 15.6	61.7 ± 14.6	< 0.0001	44.0 ± 17.4	38.6 ± 15.7	55.7 ± 14.8	< 0.0001
*< 60 years*	1386(72.2)	1089(81.1)	297(18.9)	< 0.0001	827(79.0)	619(77.1)	208(22.9)	< 0.0001
*≥ 60 years*	575(27.8)	152(29.2)	423(70.8)		279(21.0)	88(36.2)	191(63.8)	
Race								
*White only*	488(57.8)	301(65.9)	187(34.1)	< 0.0001	276(56.0)	178(68.7)	98(31.3)	< 0.0001
*Hispanics only*	725(18.4)	446(70.4)	279(29.6)		342(19.2)	227(74.4)	115(25.6)	
*Black only*	385(12.4)	229(62.0)	156(38.0)		248(12.6)	131(58.0)	117(42.0)	
Others	363(11.4)	265(69.9)	98(30.1)		240(12.2)	171(69.4)	69(30.6)	
Education								
*< High School*	1861(97.1)	1145(65.8)	716(34.2)	< 0.0001	1025(95.7)	634(67.4)	391(32.6)	< 0.0001
*≥ High School*	98(2.9)	94(97.1)	04(2.9)		80(4.3)	72(93.3)	08(6.7)	
Employed								
*No*	888(41.1)	450(51.6)	438(48.4)	< 0.0001	367(26.4)	196(61.2)	171(38.8)	< 0.0001
*Yes*	1068(58.9)	787(77.2)	281(22.8)		736(73.6)	508(70.9)	228(29.1)	
Income^a^								
*≤ $24,999*	520(18.9)	269(52.9)	251(47.1)	< 0.0001	220(13.7)	133(69.2)	87(30.8)	< 0.0001
*> $24,999*	1278(81.1)	871(70.5)	408(29.5)		794(86.3)	519(68.9)	275(31.1)	
Marital Status								
*Never married*	316(16.1)	243(83.1)	73(16.9)	< 0.0001	204(20.9)	165(82.9)	39(17.1)	< 0.0001
*Married*^b^	1089(62.7)	715(68.9)	374(31.1)		716(71.8)	435(66.3)	281(33.7)	
*Widowed*^c^	456(21.3)	187(43.4)	269(56.6)		107(7.4)	34(33.8)	73(66.2)	
Alcohol use								
*No*	609(27.4)	349(62.5)	260(37.5)	< 0.0001	238(21.7)	163(72.9)	75(27.1)	< 0.0001
*Yes*	1036(72.6)	653(66.0)	383(34.0)		737(78.3)	451(67.9)	286(32.6)	
BMI (kg/m^2^)	29.3 ± 7.5	28.0 ± 6.8	31.9 ± 8.2	< 0.0001	28.2 ± 6.8	27.0 ± 5.5	30.8 ± 6.6	< 0.0001
Normal Weight	559(33.1)	435(79.9)	124(20.1)	< 0.0001	323(30.6)	256(84.9)	67(15.1)	< 0.0001
Overweight	521(28.5)	328(66.8)	193(33.2)		323(38.2)	253(66.9)	140(33.1)	
Obese	770(38.4)	402(55.5)	368(44.5)		334(31.3)	157(54.4)	177(45.6)	
Hypertension Awareness								
*No*	1316(69.8)	1241(95.5)	75(4.5)	< 0.0001	790(75.9)	707(90.4)	83(9.6)	< 0.0001
*Yes*	645(30.2)	00(0.0)	645(100.0)		315(24.1)	00(0.0)	315(100.0)	
SBP (mmHg)	121.4 ± 17.9	114.0 ± 11.2	135.8 ± 19.9	< 0.0001	124.1 ± 15.7	117.8 ± 9.5	137.7 ± 17.6	< 0.0001
DBP (mmHg)	68.7 ± 10.6	67.9 ± 8.9	70.2 ± 13.2	< 0.0001	71.7 ± 11.6	70.0 ± 9.2	75.4 ± 15.0	< 0.0001
PSE								
*No*	1471(76.8)	928(67.1)	543(32.9)	< 0.0001	794(75.5)	484(67.3)	310(32.7)	< 0.0001
*Yes*	484(23.2)	310(65.5)	174(34.5)		311(24.5)	222(72.5)	89(27.5)	

^a^ - annual household income, ^b^ - married/living with partner, ^c^ - widowed/divorced/separated

Continuous variables are presented as mean ± standard deviation and compared using the t-test

Categorical variables are presented as n(%) and compared using the *x*^2^ test

### Prevalence of PSE exposure

**Fig. 1:**
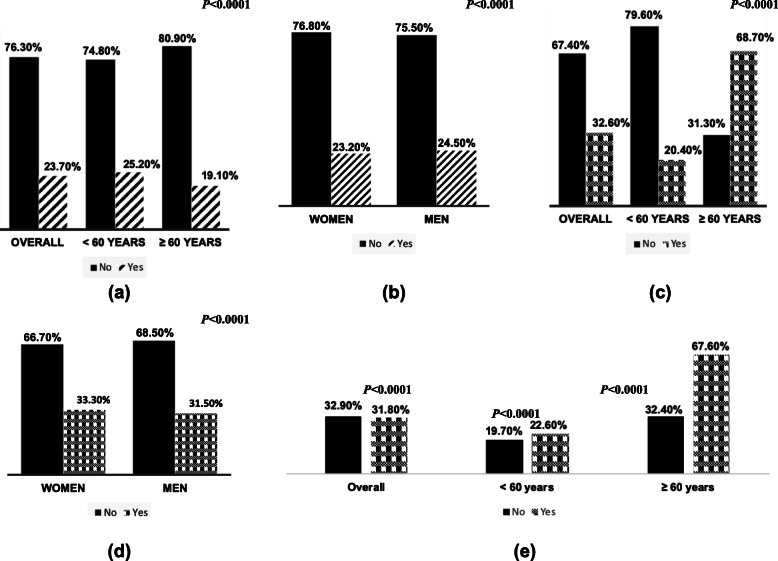
Prevalence of PSE stratified by age (**a**), and sex (**b**), prevalence of hypertension stratified by age (**c**) and sex (**d**) and prevalence of hypertension stratified by PSE (no/yes) in the overall population and by age stratification (**e**) in the 2015–2016 NHANNES Data

In all, 23.7% of the overall sample reported PSE (Fig. 1a) and prevalence of PSE was significantly higher (*P < 0.0001)* among young adults (25.2%) compared to old adults (19.1%). Also, the prevalence of PSE was significantly higher among men (24.5%) than women (23.2%) - Fig. 1b.

### Prevalence of hypertension and associated risk factors

Overall, 32.6% of the sample had hypertension (Fig. 1c). Hypertension prevalence was significantly higher among old adults (68.7%) compared to young adults (20.4%). Also, the prevalence of hypertension (Fig. 1d) was significantly higher (*P < 0.0001)* among women (33.3%) compared to men (31.5%). Hypertensive subjects (Table 1) were significantly older (59.3 ± 15.2 years) than normotensives (40.7 ± 16.6 years), the proportion of subjects with hypertension among respondents who had completed less than high school education (33.6%) was higher compared to those who had completed high school or more (4.7%), those unemployed (45.8%) presented higher prevalence of hypertensive than those employed (25.4%) and obese subjects (44.9%) were significantly more hypertensive than normal-weight subjects (18.4%). Also, Blacks (39.5%) had a higher prevalence of hypertension than whites (33.1%) or Hispanics (28.1%) and proportion of hypertensive subjects among alcoholics (33.5%) was significantly lower compared to similar subjects among non-alcoholics (34.1%). Among young adults (Table 1), men (22.9%) more than women (18.9%), respondents who are employed (20.6%) more than those unemployed (20.1%) and alcoholics (21.9%) more than non-alcoholics (18.1%) were likely to present a higher prevalence of hypertension.

Among women only (Table 2), the prevalence of hypertension was significantly higher among old adults (70.8%) compared to young adults (18.9%), but those with at least a high school education (2.9%) presented a significantly lower prevalence of hypertension compared with those with lower education (34.2%). Prevalence of hypertension was significantly higher among obese subjects (44.5%) compared to normal-weight subjects (20.1%). The result trended similarly among men, but men from a household with earning worth *>$24,*999*/annum* (31.1%) presented a significantly higher prevalence of hypertension compared to men from households with incomes *≤$24,*999*/annum* (30.8%) and prevalence of hypertension was significantly higher among men who reported using alcohol (32.6%) compared to men who do not use alcohol (27.1%).

Furthermore, we stratified hypertensive subjects only by sex (Table S1) and found hypertensive women (61.7 ± 14.6 years) were significantly older than hypertensive men (55.7 ± 14.8 years). A higher proportion of hypertensive men (0.9%) had an education not less than high school compared to hypertensive women (0.3%) and a higher proportion of men (67.6%) had a form of employment compared to women (40.3%) among hypertensive subjects only. Similarly, men (81.3%) more than women (70.7%) are likely to use alcohol among hypertensives. Contrariwise, the prevalence of obesity was significantly higher among hypertensive women (51.5%) than hypertensive men (45.2%), and level of hypertension awareness was higher among hypertensive women (90.6%) than their male counterpart (76.8%). Similarly, the prevalence of PSE was significantly higher among hypertensive women (24.0%) than hypertensive men (21.4%).

### PSE exposure and hypertension

PSE was significantly associated with hypertension status (Fig. 1e). Findings among young adults only revealed hypertension prevalence was significantly higher among subjects with PSE (22.6%) compared to those without PSE (19.7%). Similarly, we found hypertension prevalence was significantly higher among those with PSE (67.6%) compared to subjects without PSE (32.4%) among old adults.

### Adjusted odds of hypertension

In the overall population (Table 3), men were more likely at risk of hypertension than women; aOR: 1.295, 95% CI: 1.293 to 1.296 and the adjusted odds of hypertension with age was aOR: 1.080, 95% CI: 1.079 to 1.080. Respondents who are married or in partnership (aOR: 1.053, 95% CI: 1.051 to 1.055) and widows/divorced (aOR: 1.204, 95% CI: 1.201 to 1.206) were likely to be hypertensive compared to those who were never married. Alcohol users (aOR: 1.163 95% CI: 1.162 to 1.165) were likely to be hypertensives compared to non-alcohol users. Similarly, respondents who are overweight (aOR: 2.219 95% CI: 2.216 to 2.223) and obese (aOR: 3.995 95% CI: 3.990 to 4.001) were likely at risk of hypertension than normal weights subjects.

**Table 3:** Independent association of socio-demographic, lifestyle and PSE with Hypertension in the NHANNES Data

Characteristics	All		< 60 years		≥60 years	
	aOR (95%CI)	*P*	aOR (95%CI)	*P*	aOR (95%CI)	*P*
Sex						
(*Men*)	1.295 (1.293, 1.296)	< 0.0001	1.661 (1.658, 1.663)	< 0.0001	0.765 (0.764, 0.767)	< 0.0001
Age (in years)	1.080 (1.079, 1.080)	< 0.0001	1.082 (1.081, 1.082)	< 0.0001	1.036 (1.036, 1.036)	< 0.0001
Race^a^						
*(Hispanics only)*	0.522 (0.521, 0.523)	< 0.0001	0.494 (0.493, 0.495)	< 0.0001	0.680 (0.677, 0.682)	< 0.0001
*(Black only)*	0.629 (0.627, 0.630)	< 0.0001	0.564 (0.563, 0.566)	< 0.0001	0.999 (0.994, 1.004)	0.706
*(Others)*	1.298 (1.295, 1.301)	< 0.0001	1.320 (1.317, 1.323)	< 0.0001	1.302 (1.296, 1.309)	< 0.0001
Employed (*Yes*)	0.977 (0.976, 0.979)	< 0.0001	0.906 (0.905, 0.908)	< 0.0001	0.944 (0.942, 0.946)	< 0.0001
Income (*>$24,999*)	0.758 (0.757, 0.759)	< 0.0001	0.886 (0.884, 0.888)	< 0.0001	0.615 (0.614, 0.617)	< 0.0001
Marital Status^b^						
(*Married only*)	1.053 (1.051, 1.055)	< 0.0001	1.083 (1.081, 1.085)	< 0.0001	0.811 (0.807, 0.814)	< 0.0001
(*Widow/Divorced/Separated*)	1.204 (1.201, 1.206)	< 0.0001	1.379 (1.376, 1.383)	< 0.0001	0.823 (0.819, 0.827)	< 0.0001
Alcohol use (*Yes*)	1.163 (1.162, 1.165)	< 0.0001	1.186 (1.184, 1.188)	< 0.0001	1.129 (1.127, 1.131)	< 0.0001
BMI^c^						
(*Overweight*)	2.219 (2.216, 2.223)	< 0.0001	2.707 (2.702, 2.712)	< 0.0001	1.728 (1.724, 1.732)	< 0.0001
(*Obese*)	3.995 (3.990, 4.001)	< 0.0001	4.839 (4.830, 4.848)	< 0.0001	3.137 (3.130, 3.145)	< 0.0001
***PSE*** **(*Yes*)**	**1.038 (1.037, 1.040)**	**< 0.0001**	**1.095 (1.094, 1.097)**	**< 0.0001**	1.110 (1.108, 1.113)^*^	**< 0.0001**

^a^ - White only as a reference; ^b^ - never married as reference; ^c^ – normal weight as reference; *PSE* Passive smoke exposure

*Attenuated by BMI

Contrariwise, respondents of Hispanic origin (aOR: 0.522, 95% CI: 0.521 to 0.523) and Black origin (aOR: 0.629, 95% CI: 0.627 to 0.630) were less likely to be hypertensive compared to Whites. Similarly, those employed (aOR: 0.977, 95% CI: 0.976 to 0.979) were less likely to present hypertension risk than those unemployed and respondents from households with an annual income *greater than $24,999* (aOR: 0.758, 95% CI: 0.757 to 0.759) were less likely to be hypertensive compared to those from households with an annual income *less than $24,999*.

Similar results were observed independent of the sex and age stratification, but among old adults, men (aOR: 0.765, 95CI%: 0.764 to 0.767) were less likely to be hypertensive compared to women and respondents who are married (aOR: 0.811, 95CI%: 0.807 to 0.814) were less likely to be hypertensive compared to those who never married. Also (Table 4), employed men (aOR: 2.258, 95CI%: 2.253 to 2.264) were more likely at risk of hypertension compared to unemployed men, but married men (aOR: 0.674, 95CI%: 0.672 to 0.676) were less likely at risk of hypertension compared to men who are never married.

**Table 4:** Independent association of socio-demographic, lifestyle and PSE with Hypertension stratified by sex in the NHANNES Data

Characteristics	Women		Men	
	aOR (95%CI)	***P***	aOR (95%CI)	***P***
Age (in years)	1.083 (1.083, 1.083)	< 0.0001	1.083 (1.083, 1.083)	< 0.0001
Race^a^				
*(Hispanics only)*	0.500 (0.499, 0.502)	< 0.0001	0.547 (0.546, 0.549)	< 0.0001
*(Black only)*	0.608 (0.606, 0.609)	< 0.0001	0.682 (0.680, 0.684)	< 0.0001
*(Others)*	1.321 (1.317, 1.325)	< 0.0001	1.513 (1.507, 1.518)	< 0.0001
Employed (*Yes*)	0.722 (0.721. 0.723)	< 0.0001	2.258 (2.253, 2.264)	< 0.0001
Income (*>$24,999*)	0.578 (0.577, 0.579)	< 0.0001	1.248 (1.244, 1.252)	< 0.0001
Marital Status^b^				
(*Married only*)	1.174 (1.172, 1.177)	< 0.0001	0.674 (0.672, 0.676)	< 0.0001
(*Widow/Divorced/Separated*)	1.014 (1.011, 1.017)	< 0.0001	1.198 (1.910, 1.926)	< 0.0001
Alcohol use (*Yes*)	1.272 (1.270, 1.274)	< 0.0001	1.114 (1.112, 1.117)	< 0.0001
BMI^c^				
(*Overweight*)	1.837 (1.833, 1.840)	< 0.0001	3.231 (3.223, 3.239)	< 0.0001
(*Obese*)	3.353 (3.347, 3.358)	< 0.0001	5.803 (5.789, 5.818)	< 0.0001
***PSE*** **(*Yes*)**	**1.240 (1.238, 1.242)**	**< 0.0001**	**0.755 (0.754, 0.757)**	**< 0.0001**

^a^ - White only as a reference; ^b^ - never married as reference; ^c^ – normal weight as reference; *PSE* - Passive smoke exposure

### PSE and odds of hypertension

Overall (Table 3), PSE was independently associated with hypertension (aOR = 1.038, 95% CI: 1.037 to 1.040) after full adjustments for covariates. The odds of the PSE-hypertension link among young adults was, aOR: 1.095, 95CI%: 1.094 to 1.097. Furthermore (Table 4), PSE was associated with increased odds of hypertension among women (aOR: 1.240, 95% CI: 1.238 to 1.242) but not among men (aOR: 0.755, 95% CI: 0.754 to 0.757). Details of the mediation/interaction analysis using multiple regression models are presented in Table S2. Odds of hypertension among people with PSE was highest when adjusted for age; overall (aOR: 1.320, 95% CI: 1.319 to 1.322), among women (aOR: 1.585, 95% CI: 1.583 to 1.587) and young adults, < 60 years (aOR: 1.490, 95% CI: 1.488 to 1.492) and attenuated by BMI among old adults (≥60 years).

**Fig. 2:**
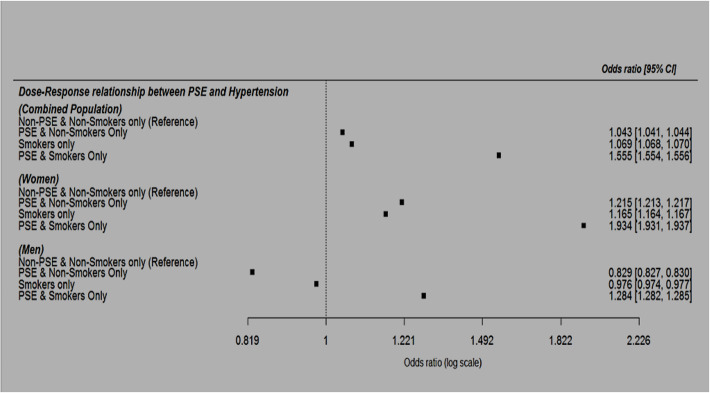
Multivariable-adjusted OR and 95%CI of the dose-response relationship between PSE and hypertension in the combined population, among women and men

Furthermore, we found PSE increased odds of hypertension in a dose-dependent manner with a non-overlapping confidence interval (Fig. 2). The aOR for hypertension risk (using non-smokers without PSE as reference) was 1.043, 95% CI: 1.041 to 1.044 among non-smokers with PSE, 1.069, 95% CI: 1.068 to 1.070 among smokers only (without PSE) and 1.555, 95% CI: 1.554 to 1.556 among smokers with PSE.

## Discussion

We presented a snippet of well-articulated evidence (a large, diverse and multicultural dataset) to demonstrate that PSE is dose-dependently associated with increased odds of hypertension and by this filling a gap in knowledge on the significance of PSE in the pathophysiology of hypertension risk taking into account age and sex differences. Our sex-stratified analysis revealed a significant interaction between PSE and hypertension. We found PSE was independently associated with increased odds of hypertension among women, but not among men.

In our report, we found PSE increased odds of hypertension which is in tandem with similar reports on the contribution of PSE to hypertension [24–28] and cardiovascular events [13, 20, 25, 29, 30]. According to similar reports from other populations, PSE independently increased the risk of hypertension by 11% in a Japanese population [24], 16% in among Korea adults [26] and 38% among Chinese women [27]. Similarly, PSE accounted for about 379,000 ischaemic heart disease-related mortality among adults worldwide in 2004 [29]. Also, PSE accounts for approximately 3million deaths and dis-ability life adjusted years among about 11million people worldwide [29]. Further to this, PSE has been suggested to be associated with increased risk of some cancers [29, 31], cognitive impairment [32], chronic kidney disease [33] and reduced coronary flow velocity reserve (CVFR) [34]. The pathophysiology of PSE and hypertension risk is multifaceted and yet to be clearly understood, but can be explained in several ways,

First, It is likely PSE impairs CVFR to promote endothelial dysfunction (ED) and consequently hypertension. A study by Otsuka et al. [34] presented evidence for PSE being associated with the slowing down of CVFR – an unswerving signal for ED [13]. ED is a function of endothelial-dependent inflammatory activation and/or debilitated vasodilation which has been linked to hypertension via multiple metabolic and vascular pathways [35]. For example, attenuated functions of endothelial-mediated dilation (regulated by nitric acid, endothelium-derived hyperpolarizing factors and prostacyclin) and constriction (regulated by endothelin-1, vasoconstrictor prostanoids, angiotensin II and superoxide anions) in regulating vascular smooth muscle tone promotes the onset of CVD such as hypertension and accelerated vascular impairment [36]. Also, ED is a risk factor for numerous pathological conditions that can lead to functional impairments in the micro-vasculature that can promote hypertension risk [37]. However, evidence(s) is still growing to support the PSE-ED-hypertension imposing the need for molecular studies to discern the PSE-hypertension relationship.

Second, nicotine (a prime and hazardous constituent of tobacco smoke) is a sympathetic stimulant of the peripheral and central nervous system to promote the release of neurotransmitters such as catecholamines [38] which may likely advance vascular damages such as elevated heart rate, blood pressure and compromised cardiac productivity [39]. Also, inhaled nicotine can be absorbed into the arteries and has the capacity to elicit acute cardiac failure depending on the quantum and velocity of absorption [38]. Similarly, nicotine-induced lipolysis can lead to increased free fatty acids and glycerol in the bloodstream and may result in upregulated fat metabolism-driven oxygen demand, increased coronary blood flow and myocardial oxygen intake [40].

Third, PSE could also promote the risk of pf hypertension through the calcification of the aortic arch. PSE has been associated with the incidence of aortic arch calcification (AAC) [41]. AAC has been implicated as a prime risk factor of coronary heart disease [41, 42] with evidence associating it with hypertension [41]. The chronic thickening of blood vessels by chemical agents from inhaled smoke can narrow the blood vessels and imposes an increase in heart pressure to supply blood thereby increasing the risk of hypertension.

Furthermore, the divergent PSE-hypertension association between women and men in our report deserves some clarifications. We found PSE was associated with increased odds of hypertension among women, but not among men. A similar observation has been documented in other reports [43–45] linking PSE with CVD and depression, and most women are likely at higher risk [46] due to exposure from smokers in vulnerable environments such as private households, bars, restaurants etc. For example, a recent report by Xu and colleagues [44] found PSE significantly increased the risk of AAC among women but not among men in China. However, Kim et al. [26] and Tamura et al. [24] in two separate studies from South Korea and Japan respectively found PSE increased the odds of hypertension independent of sex differences.

In the light of dire implications of exposure to smoke [25, 47], It would be counter-intuitive to interpret the lower likelihood of hypertension observed among men in our report as protective. It may be attributable to factors relating to hormonal differences that promote mechanistic variations in the epidemiology, pathophysiology and manifestation of CVD [48, 49]. Also, reverse causality and residual confounding are likely. Howbeit, the need for policies that promote smoke-free residential areas and apartments in addition to urban housing design that prevents smoking would be effective in downplaying PSE, particularly among non-smoking vulnerable populations [50, 51]. Also, designing family-oriented smoking guidelines are necessary to protect vulnerable women and children from PSE. In tandem with previous studies, our study observed certain risk factors such as; age, tobacco smoking [6] and BMI [12] were associated with increased odds of hypertension.

PSE prevalence in our study was analogous to findings from similar studies in other settings [19, 25, 32, 43, 52–56]. For example, the prevalence of PSE was 36.1% in 2011 [19] and 31,1% in 2019 [53] in South Korea, 38.8% in Nigeria [52] and approximately 16–39% in India [43]. Worldwide 33% and 35% of non-smoking men and women respectively had PSE in 2004 [29]. Further to this, Warren and colleagues [30] have provided evidence suggesting non-smokers are at higher risk of exposure to PSE than smokers. PSE is one of the foremost indoor pollutants globally. The primary factor promoting PSE indoors include having a member(s) of household who smoke but living conditions such as household size, natural ventilation (determined by architectural design of households and community regional planning) are secondary factors that also affects PSE and differ widely across households. Broadly speaking, the exposures and health consequences of PSE are critical and these estimates infer PSE is a life-threatening public health challenge demanding effective interventions ranging from public health policy regulation(s) to behavioural re-orientation and community planning to lessen the potential risk of diseases and disability-adjusted life years attributable to PSE.

Some limitations in this report are worth highlighting. The cross-sectional nature of this report makes it tedious to infer time-based causality. Moreover, the survey asked questions about recent PSE, making the potential links with hypertension more complicated and perhaps partly erodes objectivity in PSE evaluation. PSE is a multifactorial phenomenon depending on the intensity, time and place of exposure as well as the extent of ventilation in the environment. It is monotonous to combine these factors in a single study. However, the findings of our report are valid to make a case for public health intervention and further studies given the NHANES data used in this report is a countrywide, multicultural, large-scale population dataset suitable to test the hypothesis for this study. Also, the multi-variate adjustment for several confounding factors in this report is a promising strength to assert the findings of this report.

## Conclusions

In conclusion, PSE was dose-dependently associated with increased odds of hypertension independent of socio-cultural disparities. Aside from the need for a longitudinal multi-ethnic cohort to validate these findings, intervention strategies ranging from policy to behavioural re-orientation and eco-friendly environmental remodelling would not be out of place to obviate the tendencies of PSE in aggravating the already high burden of hypertension.

## Supplementary Information

**Additional file 1: Table S1.** Socio-demographic and metabolic characteristics Hypertensive subjects only stratified by sex in the NHANNES Data. **Table S2.** Multivariable adjusted odds ratios and 95% CI of PSE and hypertension among the non-smoking population in the 2015–2016 NHANES data.

## Data Availability

Data for this study were sourced from the 2015–2016 National Health and Nutrition Examination Survey of the United States and it is available here: https://wwwn.cdc.gov/nchs/nhanes

## Abbreviations

AAC: Aortic arch calcification
aOR: Adjusted odds ratio
BMI: Body mass index
BP: Blood pressure
CDC: Centre for disease control and prevention
CVD: Cardiovascular diseases
CVFR: Coronary flow velocity reserve
DBP: Diastolic blood pressure
ED: Endothelial dysfunction
NCHS: National Centre for Health Statistics
NHANES: National health and nutrition examination survey
PSE: Passive smoke exposure
SBP: Systolic blood pressure
US: United States
