# Supplementary material for: Passive smoking exposure and the risk of hypertension among non-smoking adults: the 2015–2016 NHANES data
Source: Clin Hypertens. 2021 Jan 1;27:1. doi: 10.1186/s40885-020-00159-7 (PMC7775627; doi:10.1186/s40885-020-00159-7)
Supplement: Supplementary file 1 — Additional file 1: Table S1. Socio-demographic and metabolic characteristics Hypertensive subjects only stratified by sex in the NHANNES Data. Table S2. Multivariable adjusted odds ratios and 95% CI of PSE and hypertension among the non-smoking population in the 2015–2016 NHANES data. [file 40885_2020_159_MOESM1_ESM.docx]

| **Table S1: Socio-demographic and metabolic characteristics Hypertensive subjects only stratified by sex in the NHANNES Data** | | | |
| --- | --- | --- | --- |
| **Characteristics** | **Women (n=720)** | **Men (n=399)** | ***P*** |
| Age (years) | 61.7±14.6 | 55.7±14.8 | <0.0001 |
| *<60years* | 297(40.9) | 208(57.4) | <0.0001 |
| *≥60years* | 423(59.1) | 191(42.6) |  |
| Race *White only* | 187(59.2) | 98(55.6) | <0.0001 |
| *Hispanics only* | 279(16.4) | 115(15.6) |  |
| *Black only* | 156(14.1) | 117(16.9) |  |
| Others | 98(10.3) | 117(11.9) |  |
| Education *<High School* | 716(99.7) | 391(99.1) | <0.0001 |
| *≥High School* | 04(0.3) | 07(0.9) |  |
| Employed *No* | 438(59.7) | 171(32.4) | <0.0001 |
| *Yes* | 281(40.3) | 228(67.6) |  |
| Income^^^ *≤ $24,999* | 251(27.1) | 87(13.6) | <0.0001 |
| *>$24,999* | 408(72.9) | 275(86.4) |  |
| Marital Status *Never married* | 73(7.9) | 39(10.9) | <0.0001 |
| *Married** | 374(57.0) | 281(74.1) |  |
| *Widowed^#^* | 269(35.1) | 73(15.0) |  |
| Alcohol use *No* | 260(29.3) | 75(18.7) | <0.0001 |
| *Yes* | 383(70.7) | 286(81.3) |  |
| BMI (kg/m^2^) | 31.9±8.2 | 30.8±6.6 | <0.0001 |
| Normal Weight | 124(20.1) | 67(14.6) | <0.0001 |
| Overweight | 193(28.4) | 140(40.1) |  |
| Obese | 368(51.5) | 177(45.2) |  |
| Hypertension Awareness *No* | 75(9.4) | 83(23.2) | <0.0001 |
| *Yes* | 645(90.6) | 315(76.8) |  |
| SBP (mmHg) | 135.8±19.9 | 137.7±17.6 | <0.0001 |
| DBP (mmHg) | 70.2±13.2 | 75.4±15.0 | <0.0001 |
| PSE *No* | 543(76.0) | 310(78.6) | <0.0001 |
| *Yes* | 174(24.0) | 89(21.4) |  |
| ^ - annual household income, * - married/living with partner, # - widowed/divorced/separated  Continuous variables are presented as mean±standard deviation and compared using the t-test.  Categorical variables are presented as n(%) and compared using the *x*^2^ test | | | |

| Table S2: Multivariable adjusted odds ratios and 95%CI of PSE and hypertension among the non-smoking population in the 2015-2016 NHANES data | | | | | | | | | |
| --- | --- | --- | --- | --- | --- | --- | --- | --- | --- |
|  | All |  | Sex | | |  | Age | | |
|  | Combined |  | Women |  | Men |  | < 60 years |  | ≥ 60 years |
|  | OR (95%CI) |  | OR (95%CI) |  | OR (95%CI) |  | OR (95%CI) |  | OR (95%CI) |
| **Crude odds** | 0.954 (0.953, 0.956) |  | 1.072 (1.071, 1.073) |  | 0.780 (0.778, 0.781) |  | 1.193 (1.191, 1.194) |  | 0.946 (0.944, 0.948) |
| ***Adjusted odds*** |  |  |  |  |  |  |  |  |  |
| Model 1 | 0.955 (0.955, 0.956) |  | na |  | na |  | 1.186 (1.185, 1.188) |  | 0.935 (0.933, 0.937) |
| Model 2 | 1.320 (1.319, 1.322) |  | 1.585 (1.583, 1.587) |  | 0.990 (0.988, 0.991) |  | 1.490 (1.488, 1.492) |  | 0.951 (0.949, 0.953) |
| Model 3 | 1.208 (1.207, 1.210) |  | 1.499 (1.497, 1.501) |  | 0.855 (0.854, 0.857) |  | 1.301 (1.299, 1.303) |  | 0.959 (0.958, 0.961) |
| Model 4 | 1.196 (1.195, 1.198) |  | 1.498 (1.496, 1.500) |  | 0.838 (0.836, 0.839) |  | 1.292 (1.291, 1.294) |  | 0.951 (0.949, 0.953) |
| Model 5 | 1.164 (1.163, 1.166) |  | 1.467 (1.465, 1.469) |  | 0.799 (0.797, 0.800) |  | 1.231 (1.229, 1.233) |  | 0.944 (0.992, 0.996) |
| Model 6 | 1.161 (1.160, 1.163) |  | 1.490 (1.488, 1.493) |  | 0.747 (0.745, 0.748) |  | 1.223 (1.221, 1.224) |  | 1.012 (1.010, 1.014) |
| Model 7 | 1.127 (1.126, 1.128) |  | 1.410 (1.408, 1.412) |  | 0.751 (0.749, 0.752) |  | 1.126 (1.125, 1.128) |  | 1.110 (1.108, 1.113) |
| Model 8 | 1.038 (1.037, 1.040) |  | 1.240 (1.238, 1.242) |  | 0.755 (0.754, 0.757) |  | 1.095 (1.094, 1.097) |  | 0.906 (0.904, 0.908) |
| Model 1; crude odds adjusted for sex  Model 2; model 1 additionally adjusted for age  Model 3: model 2 additionally adjusted for race  Model 4: model 3 additionally adjusted for employment status  Model 5: model 4 additionally adjusted for income  Model 6: model 5 additionally adjusted for marital status  Model 7: model 6 alcohol use  Model 8 (full model): model 7 additionally adjusted for BMI  Note: All odds ratios were significant at *P<0.00001*  na – not available | | | | | | | | | |
